# Editorial: Social capital and organizational citizenship behavior amongst health workforce

**DOI:** 10.3389/fpubh.2022.1089075

**Published:** 2022-12-30

**Authors:** Sima Rafiei, Amir Pakpour, Sina Abdollahzade

**Affiliations:** ^1^Social Determinants of Health Research Center, Research Institute for Prevention of Non-communicable Diseases, Qazvin University of Medical Sciences, Qazvin, Iran; ^2^Department of Neurosugery, Royal Preston Hospital, Preston, United Kingdom

**Keywords:** social capital, organizational citizenship behavior, health workforce, job satisfaction, mental health

Social capital constitutes a set of values that lets individuals work together in a team to effectively attain a common purpose. It is also considered as a set of links between organizational members, which leads to the creation of trust and interaction. As social capital resides in social relationships, these relationships act as assets representing the level of members' collective goal orientation and shared trust. These informal relationships are Organizational Citizenship behavior (OCB) which have been suggested to enhance organizational effectiveness. The absence of social capital, not only causes difficulties for the progress toward organizational development, but also it might destroy such constructive relationships. In addition, OCB has been shown to have an important impact on an organization's effectiveness, efficiency, and overall performance of organizations. Thus, the psychosocial environment of the workplace as a determinant of employee health should receive more attention in today's organizational life.

There are several researches worth mentioning here. This Research Topic aimed at widening the knowledge on social capital and organizational citizenship behavior. The issue currently includes eight papers from several fields across intangible assets and organizational citizenship behavior, organizational commitment, social capital and mental health, work-life satisfaction and social capital factors, organizational citizenship behavior and hospital performance, psychological capital and organizational citizenship behaviors, organizational trust and workplace spirituality, and resilience and social capital.

As editors of this Research Topic, it was our pleasure to review a wide range of intriguing articles within the field. In this editorial we summarize the main findings and perspectives detailed within each of the articles.

In a study by Cao et al., the influence of mental health on job satisfaction was explored through considering the mediating effect of psychological and social capital. The results signified that the positive component of mental health had a positive effect on job satisfaction, while its negative component had a negative impact on job satisfaction. Furthermore, findings revealed that psychological and social capital played a mediating role in the association between mental health and job satisfaction. Thus, authors highlighted the need for better implementation of humanistic management by nurturing employees' psychological and social capital through the mental health to enhance employees' job satisfaction.

Xu et al. also mentioned workplace social capital as respectful interactions among employees which can contribute to the formation of a wholesome psychological work environment in an organization. They also addressed transformational leadership as a style of organizational management that deals with the emotional wellbeing of workforce and inspires shared group ethics, norms, and goals. In this study with an aim to explore the influence of transformational leadership on nurses' workplace social capital, findings confirmed the significant role of the former variable on predicting nurses' workplace social capital in formation of a healthy work environment which is the foundation for efficiency and productivity of the workforce.

The effect of perceived organizational support on Organizational Citizenship Behavior (OCB) and organizational commitment in public health center was particularly significant during COVID-19 pandemic. Amid this pandemic, a study by Firmansyah et al. found a direct and significant effect between perceived organizational support and organizational commitment among nurses. In addition, a significant and positive association between organizational commitment and organizational citizenship behavior was observed. This finding highlights the fact that having high level of organizational commitment will influence how nurses are ready to work outside their job description (extra-role behavior). To develop organizational citizenship behavior and enhance the effect of perceived organizational support on organizational commitment, authors recommended improving the quality of interaction between superiors and subordinates as well as the culture and climate of the organization.

Dauner and Wilmot also mentioned state-level pre-COVID-19 pandemic social capital and contemporaneous mask policy as imperative types of social trust which can have potential effect on pandemic mental health. Authors hypothesized that social capital would confer a protective effect on population mental health level; so that during the pandemic, social trust significantly played a crucial role in lowering the levels of depression and anxiety among population.

During the COVID-19 pandemic, a research conducted by Burrmann et al. in Germany reported that higher social capital level among members of voluntary organizations remained in late 2020 despite the lockdown with its months-long restrictions of in-person meetings and interactions. However, the negative interaction effect for perceived trust in society indicated that this was not the case in every instance or will not be the case for any length of time. Therefore, the longer the pandemic lasts, the longer club activities will be disrupted and consequently the negative impacts on social capital will be more likely.

In a research by Zhang B. et al., the boundary conditions of the relationship between high-performance work systems and employee organizational citizenship behavior were explored on the China-specific management context. The research findings deepened the understanding of Human Resource Management (HRM) with a focus on organizational citizenship behavior through demonstrating that culture and identity can jointly adjust the effects of HRM on OCB. Results also emphasized that compatibility between employees' personalities and organizational values can enhance OCB in an effective manner.

In identifying the contributing factors in organizational commitment among emergency physicians, Peng et al. found the significant role of gender, age, income, frequency of daily visits, departmental promotion mechanisms, and workplace violence. They also suggested that targeted interventions are required to improve the organizational commitment of emergency physicians in an inclusive way.

Zhang W. et al. gave an overview on the association of social capital with primary healthcare (PHC) utilization of residents in China. Through analyzing data of 5,471 residents from 283 communities, authors found that one-standard deviation increase in the community social capital led to a 1.9% increase in public healthcare utilization. In fact, community social capital has been proven to play an important role in promoting PHC utilization.

## Author contributions

SR contributed to the study concept, design, and drafting of the article. AP contributed to the study design. SA contributed to revising the work critically for important intellectual content.

